# Prognostic value of microRNA-9 in cancers: a systematic review and meta-analysis

**DOI:** 10.18632/oncotarget.11466

**Published:** 2016-08-22

**Authors:** Han Sun, Yingjie Shao, Jin Huang, Siwei Sun, Yijie Liu, Pinghui Zhou, Huilin Yang

**Affiliations:** ^1^ Department of Orthopedic Surgery, The First Affiliated Hospital of Soochow University, Suzhou, 215006, P.R. China; ^2^ Department of Radiation Oncology, The Third Affiliated Hospital of Soochow University, Changzhou, 213003, P.R. China

**Keywords:** miR-9, cancer, prognosis, systematic review, meta-analysis

## Abstract

Recent studies revealed that different microRNA-9 (miR-9) expressions were associated with prognoses of different cancers. We conducted this meta-analysis to evaluate the prognostic value of miR-9. PubMed, Embase, Web of Science, and Cochrane Library (last update by November 30, 2015) were searched for literatures. A total of 17 studies from 16 articles were finally qualified and enrolled in this meta-analysis. Pooled analyses showed that a higher expression of miR-9 might predict poor overall survival (HR: 2.17, 95% CI: 1.39 – 3.41, *P* < 0.001 (7.23 * 10^−4^)), disease-free survival (HR: 5.22, 95% CI: 2.17 – 12.53, *P* < 0.001 (2.21 * 10^−4^)), and recurrence-free survival (HR: 1.57, 95% CI: 1.32 – 1.85, *P* < 0.001 (1.80*10^−7^)) in various carcinomas. However, results of subgroup analyses revealed that down-regulated miR-9 was associated with poor overall survival (HR: 0.45, 95% CI: 0.28 – 0.73, *P* < 0.001 (1.13*10^−3^)) and progress-free survival (HR: 0.46, 95% CI: 0.34 – 0.62, *P* < 0.001 (5.03*10^−7^)) in ovarian cancer patients. By subgroup analyses we also found that sample collecting time and patients’ origin had little influence on the result of OS. These results indicate that in most cancer types the highly expressed miR-9 is associated with poor survival of patients, whereas the down-regulated miR-9 may predict poor prognosis in patients with ovarian cancer.

## INTRODUCTION

Until now cancer is still the second leading cause of death, which remains a big challenge to the world [1]. Although there is an increasing trend in the number of cancer survivors that should attribute to early examination and treatment, traditional detective methods such as imaging techniques and biopsy still have their limitations [2, 3]. So it is necessary to find a new way to diagnose cancer and judge the prognosis early and precisely.

MicroRNAs (miRNAs) are a class of endogenously expressed non-coding small RNAs which contain 19 – 25 nucleotides [4]. These RNAs have been proven to play important roles in cellular growth, differentiation, proliferation, metastasis, migration, and apoptosis [4–6]. Recently, many studies have demonstrated that the regulation and expression levels of miRNAs were closely related with clinicopathological features and prognoses of cancers [7, 8]. Thus miRNAs might be used as diagnostic biomarkers for cancers.

MicroRNA-9 (miR-9) was firstly found to participate in neurogenesis as a regulator to the fate of neuronal progenitor cells [9]. This kind of miRNA has also been reported to express in various kinds of cancers [10–12]. Up to now several studies have revealed that overexpressed miR-9 was related to poor survival in some kind of carcinomas such as hepatocellular carcinoma [13], adrenocortical cancer [14], osteosarcoma [15], and breast cancer [16]. There were some opposite results as well [17–19]. Therefore, the prognostic value of miR-9 on cancer patients remained unclear. We conducted this systematic review and meta-analysis to clarify the relationship between the miR-9 expression and the survival outcome of human carcinomas.

## RESULTS

### Study characteristics

**Figure 1:**
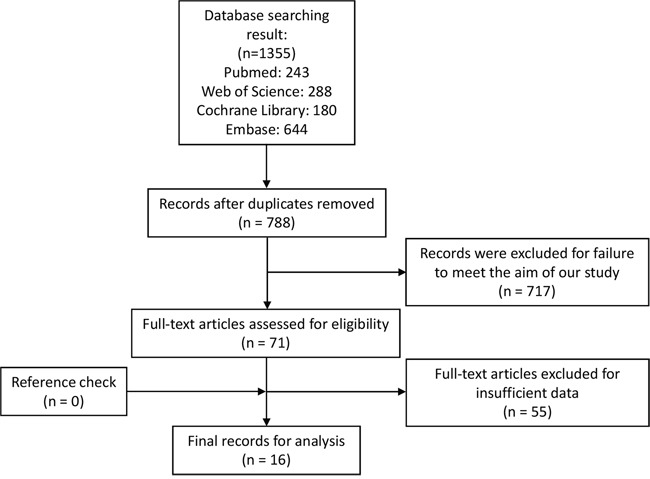
Flow chart of study selection process

By using the described searching strategy, we primarily collected 1355 records. After excluding duplicates and articles failed to meet the aim of our study, 71 records were assessed as eligible for full-text review. Then 55 full-text articles were excluded for insufficient data. Finally, 17 studies from 16 articles were qualified and enrolled in this meta-analysis [13–28]. Figure 1 revealed the flow chart of study selection process.

Main information of the included studies were summarized in Table 1. The collected 1491 patients with overall survival (OS) data, 340 patients with progress-free survival (PFS) data, 315 patient with disease-free survival (DFS) data, and 206 patients with recurrence-free survival (RFS) data were from China, German, Republic of Korea, France, Brazil, and United States. These patients were diagnosed as hepatocellular carcinoma, adrenocortical cancer, osteosarcoma, breast cancer, ovarian carcinoma, lung cancer, bladder cancer, thyroid cancer, esophageal carcinoma, glioma, and laryngeal carcinoma. Among the 17 studies, 16 measured the miR-9 expression by quantitative real-time PCR (qRT-PCR) while 1 by in situ hybridization (ISH) [20]. All studies assessed miR-9 expression in tumor tissue except 1 research in peripheral venous blood [15]. One research preferred methylated/unmethylated as cut-off values [20], one preferred positive/negative [23], one preferred mean expression level [17], one did not mention the concerning information [24], and the rests preferred median expression level. Hazard ratios (HRs) and 95% confidence intervals (CIs) were directly reported in 10 studies [13, 16, 18, 20, 22–27]. Only 1 study reported the risk ratio (RR) [25], thus we combined HRs and RR. The details of assay were summarized in Table 2. The samples in 16 outcomes were collected before any clinical treatment, in 1 outcome was collected after a period of treatment, and in 5 outcomes the related information was neglected.

**Table 1:** Characteristics of the included studies

Study ID	Country	Disease	Number	Stage	Sample	Assay	Cut-off	Survival	HR	Follow-up (months)
Cai et al. 2014 [13]	China	Hepatocellular carcinoma	200	I-IV	Tissue	qRT-PCR	Median	OS	R	Up to 60
Faria et al. 2015 [14]	Germany	Adrenocortical cancer	28 (OS) 20 (DFS)	I-IV	Tissue	qRT-PCR	Median	OS, DFS	SC	Up to 400
Fei et al. 2014 [15]	China	Osteosarcoma	118	I-III	Blood	qRT-PCR	Median	OS	SC	48 (10 - 81)
Gwak et al. 2014 [16]	Republic of Korea	Breast cancer	129 (FS) 166 (SVS)	I-III	Tissue	qRT-PCR	Median	DFS	R	68.4 (2.4 - 124.8) (FS) 93.6 (10.8 - 127.2) (SVS)
Li et al. 2013 [17]	China	Serous ovarian carcinoma	45	I-IV	Tissue	qRT-PCR	Mean	OS, PFS	SC	Up to 140 (OS) Up to 80 (PFS)
Li et al. 2015 [18]	China	Epithelial ovarian carcinoma	66	I-IV	Tissue	qRT-PCR	Median	OS, PFS	R	Up to 140 (OS) Up to 100 (PFS)
Muraoka et al. 2013 [20]	Japan	Non-small cell lung cancers	293	I-III	Tissue	ISH	Methylated/Unmethylated	OS	R	32
Pignot et al. 2013 [21]	France	Muscle-invasive bladder cancer	72	T2-4	Tissue	qRT-PCR	Median	OS, RFS	SC	30.5 (4 - 100)
Sondermann et al. 2015 [22]	Brazil	Papillary thyroid cancer	66	I-IV	Tissue	qRT-PCR	Median	RFS	R	Up to 120
Song et al. 2014 [23]	China	Esophageal squamous cell carcinoma	243	I-IV	Tissue	qRT-PCR	+/−	OS	R	Up to 60
Sun et al. 2013 [19]	China	Serous ovarian cancer	113	IIIC-IV	Tissue	qRT-PCR	Median	PFS	SC	Up to 50
Wu et al. 2013 [25]	China	Glioma	128	I-IV	Tissue	qRT-PCR	Median	OS	(RR) R	Up to 60
Wu et al. 2014 [24]	China	Laryngeal squamous cell carcinoma	103	I-IV	Tissue	qRT-PCR	NR	OS	R	24 - 60
Xu et al. 2014 [27]	China	Non-small cell lung cancer	116	I-III	Tissue	qRT-PCR	Median	OS, PFS	R	36 (20 – 48)
Xu et al. 2014 [26]	China	Osteosarcoma	79	I-III	Tissue	qRT-PCR	Median	OS	R	Up to 60
Zhou et al. 2012 [28]	USA	Breast cancer	68	0-IV	Tissue	qRT-PCR	Median	RFS	SC	77.5

**HR**, hazard ratio; **FS**, the first set, in which samples were from Seoul National University Bundang Hospital from 2003 to 2011; **SVS**, the second validation set, in which samples were from Seoul National University Bundang Hospital from 2003 to 2006; **ISH**, in situ hybridization; **NR**, not reported; **OS**, overall survival; **PFS**, progress-free survival; **DFS**, disease-free survival; **RFS**, recurrence-free survival; **RR**, risk ratio; **R**, reported; **SC**, survival curve.

**Table 2:** Details of HRs and their 95% CIs

Studie ID	Disease	Outcome	HR	95% CI and *P* value	Univariate analysis or multivariate analysis	Assay	Internal reference	Before or after treatment
Cai et al. 2014 [13]	Hepatocellular carcinoma	OS	4.28	2.77 – 7.23, *P* < 0.001	Multivariate analysis	qRT-PCR	U6	Before
Faria et al. 2015 [14]	Adrenocortical cancer	OS	4.45	1.02 – 19.44, *P* = 0.01	Univariate analysis	qRT-PCR	β-actin, β-glucoronidase	Unknown
Faria et al. 2015 [14]	Adrenocortical cancer	DFS	7.25	1.07 – 49.20, *P* = 0.01	Univariate analysis	qRT-PCR	β-actin, β-glucoronidase	Unknown
Fei et al. 2014 [15]	Osteosarcoma	OS	2.93	1.52 – 5.63, *P* = 0.02	Univariate analysis	qRT-PCR	U6	Before
Gwak et al. 2014 [16]	Breast cancer	DFS (FS)	12.204	1.788 – 83.299, *P* = 0.011	Multivariate analysis	qRT-PCR	U6	Before
Gwak et al. 2014 [16]	Breast cancer	DFS (SVS)	3.418	1.083 – 10.783, *P* = 0.036	Multivariate analysis	qRT-PCR	U6	Before
Li et al. 2013 [17]	Serous ovarian carcinoma	OS	0.53	0.28 – 1.00, *P* = 0.021	Univariate analysis	qRT-PCR	U6	Before
Li et al. 2013 [17]	Serous ovarian carcinoma	PFS	0.54	0.29 – 1.00, *P* = 0.0261	Univariate analysis	qRT-PCR	U6	Before
Li et al. 2015 [18]	Epithelial ovarian carcinoma	OS	0.37	0.18 – 0.76, *P* = 0.007	Multivariate analysis	qRT-PCR	U6, GAPDH	Before
Li et al. 2015 [18]	Epithelial ovarian carcinoma	PFS	0.24	0.12 – 0.50, *P* = 0.000	Multivariate analysis	qRT-PCR	U6, GAPDH	Before
Muraoka et al. 2013 [20]	Non-small cell lung cancers	OS	4.2	1.2 – 27.0, *P* = 0.026	Multivariate analysis	ISH	NR	Unknown
Pignot et al. 2013 [21]	Muscle-invasive bladder cancer	OS	3.32	1.77 – 6.20, *P* = 0.025	Univariate analysis	qRT-PCR	U6B, RNU44	Unknown
Pignot et al. 2013 [21]	Muscle-invasive bladder cancer	RFS	2.51	1.32 – 4.75, *P* = 0.025	Univariate analysis	qRT-PCR	U6B, RNU44	Unknown
Sondermann et al. 2015 [22]	Papillary thyroid cancer	RFS	1.48	1.24 – 1.77, *P* < 0.001	Univariate analysis	qRT-PCR	RNU48	Before
Song et al. 2014 [23]	Esophageal squamous cell carcinoma	OS	1.543	1.112 – 2.140, *P* = 0.009	Multivariate analysis	qRT-PCR	U6, GAPDH	Before
Sun et al. 2013 [19]	Serous ovarian cancer	PFS	0.53	0.36 – 0.79, *P* = 0.01	Univariate analysis	qRT-PCR	U6	After
Wu et al. 2013 [25]	Glioma	OS	3.62 (RR)	1.81 – 7.33, |*P* = 0.01	Multivariate analysis	qRT-PCR	RNU6B	Before
Wu et al. 2014 [24]	Laryngeal squamous cell carcinoma	OS	3.18	2.19 – 11.91, *P* = 0.012	Multivariate analysis	qRT-PCR	U6	Before
Xu et al. 2014 [27]	Non-small cell lung cancer	OS	1.491	1.089 – 2.042, *P* = 0.013	Multivariate analysis	qRT-PCR	U6	Before
Xu et al. 2014 [27]	Non-small cell lung cancer	PFS	1.544	1.174 – 2.055, *P* = 0.002	Multivariate analysis	qRT-PCR	U6	Before
Xu et al. 2014 [26]	Osteosarcoma	OS	4.77	2.86 – 5.91, *P* = 0.002	Multivariate analysis	qRT-PCR	U6	Before
Zhou et al. 2012 [28]	Breast cancer	RFS	2.67	1.07 – 6.66, *P* = 0.08	Univariate analysis	qRT-PCR	U6	Before

**OS**, overall survival; **PFS**, progress-free survival; **DFS**, disease-free survival; **RFS**, recurrence-free survival; **FS**, the first set, in which samples were from Seoul National University Bundang Hospital from 2003 to 2011; **SVS**, the second validation set, in which samples were from Seoul National University Bundang Hospital from 2003 to 2006; **HR**, hazard ratio; **RR**, risk ratio; **ISH**, in situ hybridization; **NR**, not reported.

### Meta-analysis results

**Figure 2:**
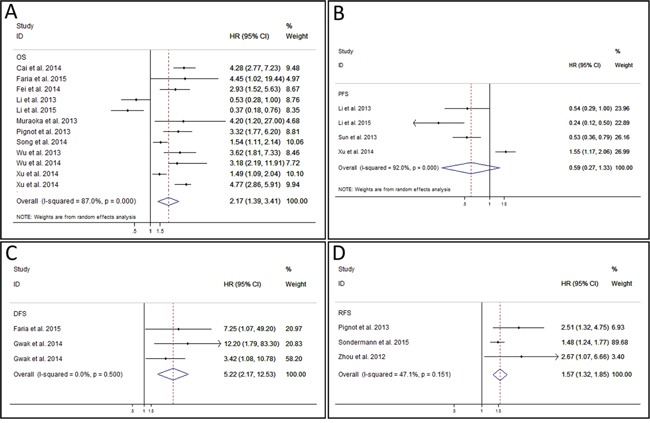
Forest plot of overall survival analysis, progress-free survival analysis, disease-free survival analysis, and recurrence-free survival analysis **A.** meta-analysis of miR-9 expression and overall survival in various cancers. **B.** meta-analysis of miR-9 expression and progress-free survival in various cancers. **C.** meta-analysis of miR-9 expression and disease-free survival in various cancers. **D.** meta-analysis of miR-9 expression and recurrence-free survival in various cancers.

A total of 12 studies reported the OS of patients [13–15, 17, 18, 20, 21, 23–27]. As the result of meta-analysis exhibited obvious heterogeneity (*P* < 0.001 (2.07 * 10^−13^), I^2^ = 87.0%), the random effect model was used to calculate the pooled HR and its 95% CI. The result revealed that higher expression of miR-9 might predict poor OS in various carcinomas, and the pooled HR was 2.17 (95% CI: 1.39 – 3.41, *P* = 0.001) (Figure 2A). PFS was reported by 4 studies [17–19, 27]. The *P* value and I^2^ of heterogeneity test were < 0.001 (3.84 * 10^−8^) and 92.0% respectively. After using random effect model, the pooled HR was 0.59 (95% CI: 0.27 – 1.33, *P* = 0.205), indicating no significant relationship between miR-9 expression and PFS (Figure 2B). The analysis result of DFS reported by 3 studies in 2 articles [14, 16] showed that the high level of miR-9 expression was related to poor DFS (HR: 5.22, 95% CI: 2.17 – 12.53, *P* < 0.001 (2.21 * 10^−4^)) (Figure 2C). The fixed effect model was used as no heterogeneity was found (*P* = 0.500, I^2^ = 0.0%). The outcomes of RFS reported by 3 studies were also pooled and analyzed [21, 22, 28]. The result of heterogeneity test was *P* = 0.151 and I^2^ = 47.1%. So, a fixed effect model was used to calculate the pooled HR which was 1.57 (95% CI: 1.32 – 1.85, *P* = 0.004) (Figure 2D). This result implied that the expression level of miR-9 was negatively correlated with the RFS of carcinomas.

**Figure 3:**
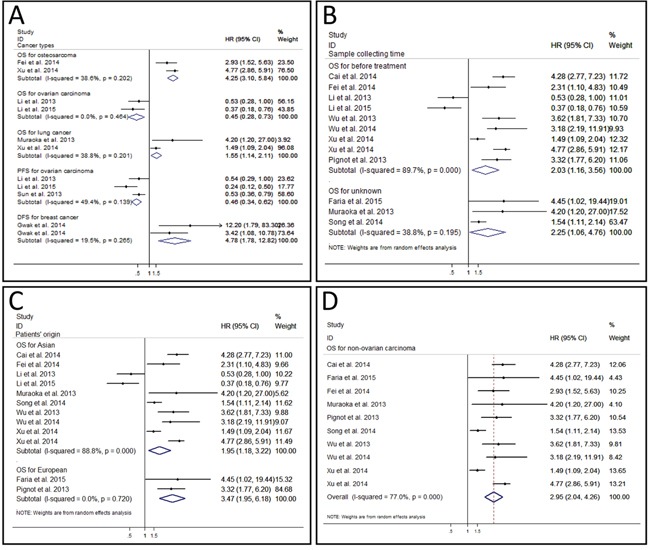
Forest plot of subgroup analysis **A.** subgroup analysis of different cancer types. **B.** subgroup analysis of sample collecting time. **C.** subgroup analysis of patients’ origin. **D.** subgroup analysis of overall survival for non-ovarian carcinoma.

Subgroup analyses were carried out in osteosarcoma, ovarian carcinoma, lung cancer, and breast cancer (Figure 3A). In part of OS, results of osteosarcoma, ovarian carcinoma, and lung cancer were pooled respectively. No significant heterogeneity was found in each group (osteosarcoma: *P* = 0.202 and I^2^ = 38.6%; ovarian carcinoma: *P* = 0.464 and I^2^ = 0.0%; lung cancer: *P* = 0.201 and I^2^ = 38.8%), thus fixed effect model was used to pool the HRs according to the carcinoma. The results of analyses revealed that the over-expressed miR-9 predicted poor OS for patients with osteosarcoma (HR: 4.25, 95% CI: 3.10 – 5.84, *P* < 0.001 (3.89 * 10^−19^)) [15, 26] and lung cancer (HR: 1.55, 95% CI: 1.14 – 2.11, *P* = 0.005) [20, 27] while predicted good OS for patients with ovarian carcinoma (HR: 0.45, 95% CI: 0.28 – 0.73, *P* = 0.001) [17, 18]. In 3 articles which used PFS to assess the outcome of ovarian carcinoma [17–19], no significant heterogeneity was found (*P* = 0.139, I^2^ = 49.4%), and the fixed model calculation produced the pooled HR as 0.46 (95% CI: 0.34 – 0.62, *P* < 0.001 (5.03*10^−7^)), indicating the significant relationship between low tissue miR-9 level and poor PFS in ovarian carcinoma. No significant heterogeneity (*P* = 0.265, I^2^ = 19.5%) was revealed in pooled result of 2 studies from one article in which DFS was used as outcome assessment value of breast cancer [16]. The combined HR reached 4.78 (95% CI: 1.78 – 12.82, *P* = 0.002) after fixed effect model was used. Thus, the over-expressed miR-9 predicted poor outcome of breast cancer patients.

We also evaluated the influences of sample collecting time (Figure 3B) and patients’ origin (Figure 3C) in OS group by the subgroup analysis. A total of 9 studies collected samples and evaluated the relationship between miR-9 expression and OS before treatment [13, 15, 17, 18, 21, 24–27], and the combined HR was 2.03 (95% CI: 1.16 – 3.56, *P* = 0.014). As obvious heterogeneity was found (*P* < 0.001 (1.35 * 10^−13^), I^2^ = 89.7%), a random effect model was used. Results of 3 studies that failed to report the exact sample collecting time in OS group were pooled as well [14, 20, 23]. The combined HR was 2.25 (95% CI = 1.06 – 4.76, *P* = 0.034), which was comparable with the pooled result of untreated patient and OS for all. On the other hand, we performed subgroup analysis of OS according to the different patients’ origins. Patients in 10 studies were from Asia [13, 15, 17, 18, 20, 23–27]. The analysis revealed that over-expressed miR-9 was associated with unfavorable OS in Asian patients (HR: 1.95, 95% CI: 1.18 – 3.22, *P* = 0.009) after a random effect model was used (*P* < 0.001 (1.48 * 10^−13^), I^2^ = 88.8%). The HRs of OS in patients from Europe were also combined [14, 21]. The pooled HR was 3.47 (95% CI: 1.95 – 6.18, P < 0.001 (2.34 * 10^−5^)), which was comparable to that of Asian patients and OS for all. These results suggested that nether the sample collecting time nor the patients’ origin had significant influence on the predictive effect of miR-9.

### Heterogeneity analysis

We used different methods to analyze the potential sources of the heterogeneity.

**Figure 4:**
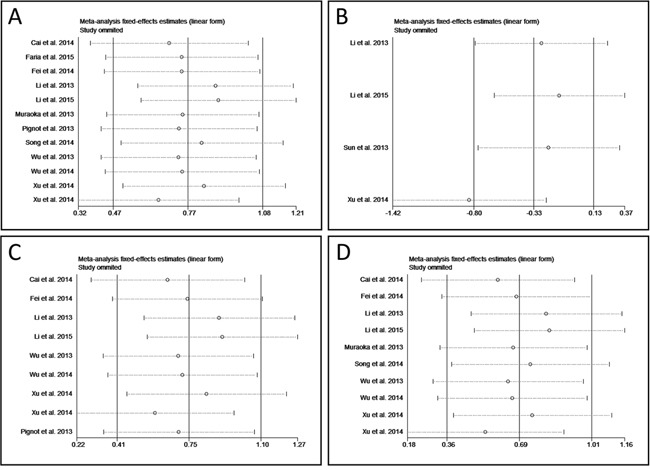
Forest plot of sensitivity analysis **A.** sensitivity analysis of overall survival. **B.** sensitivity of progress-free survival. **C.** sensitivity analysis of OS for before treatment. **D.** sensitivity analysis of OS for Asian.

A meta-regression analysis was conducted to evaluate the possible factors related to the heterogeneity of OS. Results showed that the cut-off value (*P* = 0.911), follow-up period (*P* = 0.340), risk evaluation method (*P* = 0.228), sample size (*P* = 0.444), sample specimen (*P* = 0.938), univariate analysis or multivariate analysis (p = 0.759), publication year (*P* = 0.559), and stages of cancers (*P* = 0.345) contributed little to the heterogeneity. Sensitivity analysis could help to evaluate the credibility and stability of heterogeneity by omitting each study by turns. The result showed that no individual study could significantly influence the combined HR (Figure 4A). Subgroup analyses were also carried out. We divided the 12 studies into 3 groups in accordance with the cancer type: osteosarcoma including 2 studies [15, 26], ovarian carcinoma including 2 studies [17, 18], and lung cancer including 2 studies [20, 27] (Figure 3A). No obviously heterogeneity was found in each subgroup, so the cancer type could partly explain the heterogeneity of OS analysis. However, when we performed subgroup analyses based on the sample collecting time and the patients’ origin, the heterogeneity was still significant (Figure 3B and 3C), so these two factors could hardly solely explain the heterogeneity in OS analysis group. What's more, as the studies of ovarian tumor showed opposite prognostic effect of miR-9 expression, we also carried out the subgroup analysis of OS by excluding the 2 ovarian tumor studies. Result showed that although the heterogeneity was decreased compared with that of OS for all, obvious heterogeneity could still be found (*P* < 0.001 (1.08 * 10^−5^), I^2^ = 77.0%) (Figure 3D).

If less than ten studies were included in an analysis, the meta-regression analysis is not proper to find the sources responsible for the heterogeneity. Thus, sensitivity analysis was performed in PFS group, OS for before treatment, and OS for Asian instead. Result exhibited that study of Xu et al. was responsible for the heterogeneity of PFS group (Figure 4B) [27], while in the other two subgroups the results of association between miR-9 expression and OS were relatively credible and stable (Figure 4C and 4D).

### Publication bias

**Figure 5:**
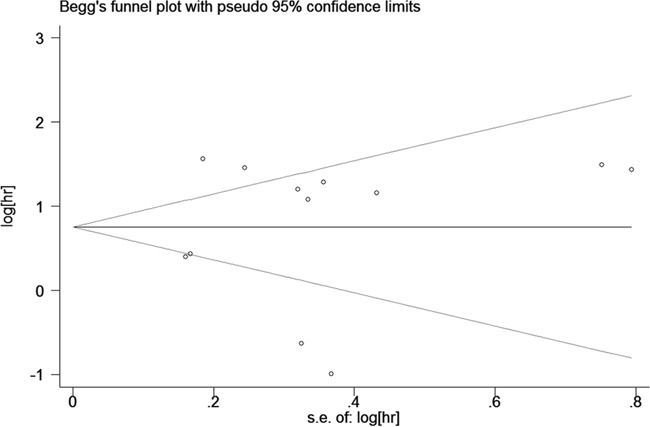
Begg's funnel plot for publication bias in overall survival

We used funnel plots and Egger's tests to evaluate the publication bias of included studies. The funnel plot of OS analysis was revealed in Figure 5, and the *P* value of Egger's regression intercept was 0.860, indicating that no evidence of significant publication bias was found in this meta-analysis. We did not analyze the publication bias and funnel plots of PFS group, DFS group, RFS group and subgroups as the number of included studies was limited and the results were not reliable.

## DISCUSSION

MiR-9 has been found to take part in the development of the nervous system and hepatocytes under physiologic conditions [29, 30], and induce the negative regulation of the acute responses in innate immunity [31]. Studies revealed that miR-9 also plays a pivotal role in tumorigenesis and tumor progression [32–34]. In some tumors such as ovarian cancer [18], colon cancer [12], esophageal cancer [35], and neuroblastoma [36], miR-9 is down-regulated and acts as the tumor suppressor. On contrary, miR-9 is up-regulated to enhance the growth and metastasis of breast cancer [37], non-small cell lung cancer [27], and so on.

Human miR-9 has been mapped to three chromosomal locations in the human genome (chromosomes1, 5, and 15), and three members are involved in miR-9 family: miR-9-1, miR-9-2, and miR-9-3 [38, 39]. Studies found that the alteration for any of these three independent genes could lead to the dysregulation of miR-9, while the differential transcription of primary miR-9 transcripts could mainly attribute to cell type and cellular context [40]. Study of Davila et al. revealed that Mef2C could activate miR-9-2 which resulted in the increased expression of miR-9 and further facilitated the neurogenic differentiation of neural progenitor cells [41]. On the contrary, miR-9-2 could be inhibited by REI-silencing transcription factor (REST) in undifferentiated neuroblastoma cells, leading to a suppression of miR-9 [42]. Shan et al. found that during hypoxia, the increased regulation of miR-9 was accompanied with the up-regulated transcription of miR-9-1 and miR-9-3 in a hypoxia-inducible factor-1α-dependent manner [40]. It is noteworthy that despite these different primary miRNA transcripts, all the three loci will give rise to the same mature miRNA sequence [43].

It is an interesting phenomenon that miR-9 shows low expression in neuroblastoma [36] and medulloblastoma [44] but high expression in glioma [25]. Zhang et al. found that down-regulated miR-9 was mainly associated with the invasion, metastasis and angiogenesis of neuroblastoma cells [36]. The same year, Annibali et al. found that low expression of miR-9 could up-regulate transcription factor inhibitor of DNA binding-2 (ID-2) to promote proliferation and inhibit differentiation of neuroblastoma cells. Their study also revealed that miR-9 could directly targets REST which served as a secondary inhibitor to prevent transition from progenitor cells to neurons. So they considered miR-9 as a competing endogenous RNA which could mediate the communication between ID2 and REST mRNAs [45]. As to medulloblastoma, its proliferation can also be promoted by the knockdown of miR-9 [44]. In glioma, highly expressed miR-9 plays dual roles of both proliferation-inhibitor and migration-enhancer, which is balanced by cyclic AMP response element-binding protein to carry out the migration-proliferation dichotomy [46]. Considering the histogenesis of these neural tumors, and the differentiation of neuroblastoma and medulloblastoma cells is inhibited by the down-regulated miR-9, the stage-specific expression of miR-9 may play a more important role besides the expression level itself. However, more in-depth studies are still needed to demonstrate this theory.

Recent studies have also found that miR-9 had opposing effects in the prognosis of different cancers [13, 14, 17, 18]. However, to our knowledge, the relationship between the miR-9 expression and the prognosis of various cancers hasn't been systematic reviewed and investigated. Thus we conducted this meta-analysis to evaluate the prognostic value of miR-9.

In this meta-analysis, a total of 16 studies with 11 different types of cancers were enrolled and 4 survival assessment parameters (OS, PFS, DFS, and RFS) were measured. The result of analysis revealed that elevated miR-9 expression did predicted poor OS in carcinomas patient. We also carried out subgroup analysis of OS to try to eliminate heterogeneity and find out the specific relationship between the miR-9 expression and the OS of each tumor. Results showed that the association between the over-expression of miR-9 and poor OS was more prominent in osteosarcoma and lung cancer patients. Conversely, in patients of ovarian carcinoma, a down-regulated miR-9 usually indicated the unsatisfactory OS. Our study found that the high expression of miR-9 might predict good PFS for cancer patients. However, the *P* value was not statistically significant (*P* = 0.205) and obviously heterogeneity was found. The result of sensitivity analysis revealed that study of Xu et al. was responsible for the heterogeneity [27]. This might because the cancer type in their study was lung cancer while in the other 3 studies was ovarian cancer. This conjecture was confirmed by the subgroup analysis as no obviously heterogeneity was found after excluding the study of Xu et al., and statistically significant was revealed between the low expression of miR-9 and poor PFS in ovarian cancer patients (*P* < 0.001 (5.03*10^−7^)). We also found that the down-regulated miR-9 was related to the good outcomes of DFS and RFS. Thus, miR-9 may be a potential predictor of poor survival in cancers except for ovarian cancer. Sun et al. attributed this result to that miR-9 could mediate the down-regulation of BRCA1 which predicted good prognosis of ovarian and could inhibit the reparation of DNA damage in ovarian cancer [19]. MiR-9 can also improve the efficacy of chemotherapeutics to ovarian cancer [18].

Therapies such as chemotherapy and radiotherapy can kill cancer cells and may further influence the miR-9. The expression of miR-9 in the included studies were detected in samples of different treatment status, such as before treatment, after treatment, and unclear. So we carried out subgroup analysis to evaluate the influence of sample collecting time. In OS group the pooled results revealed that the prognostic effect of miR-9 was comparable among the untreated group, unclear group, and OS for all group. As to PFS group, DFS group, and RFS group the data were insufficient for subgroup analysis. Our result preliminarily demonstrated that there was no significant treatment effect on the relationship between miR-9 expression and OS. Similarly, we also explored the influence of patients’ origin. Most patients in OS group were from China, and the rest were from different countries respectively, which made it hard to form subgroups based on countries strictly. So we combined the patients’ origins according to the continent each country belongs to. It was hard to carry out the subgroup analysis in other survival groups because of the limited number of included studies. The result of analysis showed that OS for all patients, OS for Asian patients, and OS for European patients shared the similar combined HRs and *P* values, initially indicating that miR-9 held comparable predicting effect of OS in different ethnicities.

Some limitations of this study should be acknowledged. First, as only 16 articles with 17 studies were included, the data in some analyses and subgroup analyses were relatively insufficient. Second, the cut-off value used in each study was different so that a clear threshold could not be set up. Additionally, different detection methods, tumor types, follow-up period, and sample sources may also affect the effectiveness and contribute to the heterogeneity. Finally, parts of HRs were calculated based on the data extracted from the survival curves, which might lead to small statistical errors.

Our meta-analysis suggests that in most cancer types the highly expressed miR-9 is associated with poor survival of patients, whereas in patients with ovarian cancer the down-regulated miR-9 may predict poor prognosis. In conclusion, miR-9 is a potentially suitable prognostic biomarker in cancers. However, considering the limitations of the current analysis, the conclusion should be cautiously interpreted. More clinical investigations with high quality and large sample size are needed to further testify the prognostic roles of miR-9 expression in cancers.

## MATERIALS AND METHODS

Guidelines of Preferred Reporting Items for Systematic Review and Meta-Analyses (PRISMA) [47] and Meta-analysis of Observational Studies in Epidemiology group (MOOSE) [48] were totally followed to carry out this meta-analysis.

### Search strategy

We carefully searched online databases including PubMed, Embase, Web of Science, and Cochrane Library (last update by November 30, 2015) for literatures. Key terms used for database research were: miR-9, cancer, carcinoma, tumor, and neoplasm. These keywords were combined by Boolean operators of “AND” and “OR”. We did not set any advanced limitations when searching the database. The references of full-text articles were also manually searched to avoid omitted studies. Two reviewers independently conducted the search. Any disagreement was unified by discussion.

### Inclusion and exclusion criteria

Literatures were considered to be eligible if they met the following criteria: (1) the subject of the study should be patients with any type of carcinoma; (2) the expression of miR-9 was measured in cancer tissue or serum; (3) the relationship between miR-9 expression level and survival outcome should be investigated. Articles were excluded based on the following criteria: (1) reviews, letters, comments, or laboratory studies; (2) miR-9 were not expressed in any cell line of the cancer; (3) investigation of a set of miRNAs rather than miR-9 alone; (4) only relationship between each miR-9 family member and prognostic outcome but lack of the result of integration; (5) studies of nondichotomous miR-9 expression levels; (6) absence of key information of survival outcome or cannot estimate HRs and 95% CIs by the shown data.

### Quality assessment

We evaluated the included studies according to the critical review checklist of the Dutch Cochrane Centre proposed by MOOSE [48]. The basic standards were as following: (1) enough report of study population; (2) clear method of study design; (3) enough report of the cancer; (4) clear definition of outcome assessment; (5) enough description of miR-9 measurement; (6) enough period of follow-up. Studies that failed to contain these seven points were excluded.

### Data extraction

Two reviewers extracted the data independently. Relevant parameters included first author's surname, year of publication, country of origin, tumor type, tumor stage, sample type and number, method, cutoff value, follow-up period, source of miRNA, sample collecting time, and HRs of miR-9 expression for OS, PFS, DFS, and RFS, as well as their 95% CIs and *P* values. If the HR and 95% CI were not reported directly, we calculated them through the total observed death events and the numbers of patients in each group reported in articles. If only Kaplan-Meier curves were available, data were extracted from graphical survival plots to estimate the HRs [49]. If a study reported the results of univariate and multivariate analysis at the same time, only the latter was extracted. This is because results of multivariate analysis were more precious due to its accounting for confounding factors. Discrepancies about data extraction were resolved by discussion among the first three authors.

### Statistical analysis

We used HRs with their corresponding 95% CIs to calculate pooled data. Statistically significant was defined as *P* < 0.05 and overall 95% CI did not include ‘1′. We use the Q test and I^2^ statistic to evaluate heterogeneity. A random effect model was used if *P* < 0.05 or I^2^ > 50% which indicated heterogeneity. On the contrary, a fixed effects model was used when *P* ≥ 0.05 and I^2^ ≤ 50%. Sensitivity analysis was used to investigate the source of heterogeneity. We used subgroup analysis, sensitive analysis, or meta regression to find out the factors contributed to heterogeneities. Publication bias was analyzed by funnel plot and Egger test. All these analyses were carried out by Stata 12.0 (Stata Corporation, College Station, TX, USA).
